# Biodiversity Loss Threatens the Current Functional Similarity of Beta Diversity in Benthic Diatom Communities

**DOI:** 10.1007/s00248-020-01576-9

**Published:** 2020-08-29

**Authors:** Leena Virta, Janne Soininen, Alf Norkko

**Affiliations:** 1 Department of Geosciences and Geography, University of Helsinki, PO Box 64, FIN-00014 Helsinki, Finland; 2 Tvärminne Zoological Station, University of Helsinki, J.A. Palméns väg 260, FI-10900 Hangö, Finland; 3 Baltic Sea Centre, Stockholm University, Stockholm, Sweden

**Keywords:** Biodiversity loss, Functional beta diversity, Microphytobenthos, Diatoms, Estuary, Freshwater marine gradient

## Abstract

The global biodiversity loss has increased the need to understand the effects of decreasing diversity, but our knowledge on how species loss will affect the functioning of communities and ecosystems is still very limited. Here, the levels of taxonomic and functional beta diversity and the effect of species loss on functional beta diversity were investigated in an estuary that provides a naturally steep environmental gradient. The study was conducted using diatoms that are among the most important microorganisms in all aquatic ecosystems and globally account for 40% of marine primary production. Along the estuary, the taxonomic beta diversity of diatom communities was high (Bray-Curtis taxonomic similarity 0.044) and strongly controlled by the environment, particularly wind exposure, salinity, and temperature. In contrast, the functional beta diversity was low (Bray-Curtis functional similarity 0.658) and much less controlled by the environment. Thus, the diatom communities stayed functionally almost similar despite large changes in species composition and environment. This may indicate that, through high taxonomic diversity and redundancy in functions, microorganisms provide an insurance effect against environmental change. However, when studying the effect of decreasing species richness on functional similarity of communities, simulated species loss to 45% of the current species richness decreased functional similarity significantly. This suggests that decreasing species richness may increase variability and reduce the stability and resilience of communities. These results highlight the importance of high taxonomic biodiversity for the stable functioning of benthic communities.

**Electronic supplementary material:**

The online version of this article (10.1007/s00248-020-01576-9) contains supplementary material, which is available to authorized users.

## Introduction

The global biodiversity crisis has increased the need for understanding the effects of biodiversity loss to ecosystem functioning and the services that ecosystems provide to humans. There is substantial evidence that taxonomically and functionally diverse communities support ecosystem functioning [e.g., 1]. However, despite evidence that diversity is threatened by environmental change [e.g., 2], our knowledge on whether the functions of communities will be affected by species loss is still very limited, especially in oceans and seas [3].

Here, a real-world study on the taxonomic and functional beta diversity of benthic diatom communities and the effects of species loss on the functional beta diversity is presented. While theory development on this subject is gaining increasing traction, real-world examples supporting these ideas are still lagging behind. Spatial beta diversity, i.e., the change in species composition between sites, is a key factor for understanding the variability in ecosystem functioning. It may be as important as alpha diversity, i.e., diversity at a single site which is a much more frequently studied variable, also for biodiversity conservation perspectives, such as designing effective protective area networks or understanding processes that maintain diversity [4]. However, most studies concentrate only on taxonomic beta diversity, which ignores the different functions that species have in the ecosystem and decreases the opportunity of finding general rules in community ecology, such as links between diversity and ecosystem functioning [5]. By considering functional beta diversity, comparisons among broad geographic regions can be made despite differences in taxonomic compositions. This can also provide new insights on the role of functional biodiversity.

In a time of accelerating global change, functional beta diversity is also of interest due to its strong relation to ecosystem functioning and services [6], as well as ecosystem stability and resilience against species loss [7]. Stable and resilient ecosystems and communities are able to retain functioning despite changing environment and consequent decreasing species richness or changing community composition [8]. Stability and resilience stem from high taxonomic and functional diversity and redundancy that decrease the effect of species loss on the functioning of the ecosystem, because several species of the community perform similar functions [7]. Although stability and resilience have usually been related to the alpha diversity of communities, they are also closely connected to beta diversity, particularly through the connectivity of communities [8]. Systems with low beta diversity, i.e., highly similar communities along the gradient, are often effectively inter-connected. In such systems, the taxonomic or functional richness or composition of a single local site is not highly different from the richness or composition of the regional species or trait pool. Due to the effective connectivity, such systems are resilient against environmental change and have a high recovery potential from disturbance [9]. Thus, beta diversity can provide information on the consequences of biodiversity loss to ecosystem resilience.

However, studying the relationship between functional beta diversity and ecosystem functioning, stability, or resilience requires careful choice of functional traits [10]. The chosen traits should not only include response traits, i.e., traits that address species’ environmental requirements (Grinnellian niche), but rather effect traits, i.e., traits that also consider resource availability, disturbances and biotic interactions and hence indicate species’ effects on the environment (Eltonian niche) [10]. In this study, traits used to determine the functional beta diversity of diatom communities include morphological traits that are closely linked to ecosystem functioning, such as biomass production and trophic webs. For example, diatom species with a morphologically low-profile growth form persist at low nutrient levels where species with high-profile or motile growth forms are unable to live [11]. Thus, low-profile species support stable biomass production also in unfavorable conditions. On the other hand, grazers are dependent on diatom species with a high-profile growth form, whereas diatom species that are able to form colonies may be resistant to grazing [12]. Hence, the trait composition of diatom communities affects the ability of benthic fauna to utilize diatoms for nutrition and, thus, has an impact on higher trophic levels.

So far, most studies on functional beta diversity have focused on large organisms [e.g., 13, 14] and studies on microorganisms, the most diverse group of life on Earth and the basis for all ecosystem functioning, are rare [but see 15, 16]. Microorganisms often differ from macroorganisms in distribution and function, which means that we cannot generalize previous results on functional beta diversity to apply to microorganisms. Diatoms are one of the most important groups of microorganisms in all aquatic ecosystems. They account globally for 40% of marine primary production [17], and their functional alpha diversity is tightly linked to ecosystem productivity [18].

This study was conducted in an estuarine environment, which represents a system with an exceptionally steep environmental gradient stretching from a freshwater river to a brackish-water archipelago and finally to the open sea. Encompassing such an area with steep environmental gradients provides an excellent platform for exploring patterns of beta diversity due to the potential for high turnover of organisms [19]. Estuaries are also one of the most heavily impacted environments and experience high human pressure, which severely threatens biodiversity and increases the importance of resolving biodiversity patterns of these valuable ecosystems.

The specific research questions and hypotheses were the following: (1) *Which are the most important environmental variables driving the taxonomic and functional beta diversity in benthic diatom communities?* Salinity was expected to be the strongest controlling factor [20]. (2) *What is the level of taxonomic and functional beta diversity of benthic diatoms in an area with strong environmental gradients?* Moderate or high taxonomic beta diversity was expected to be found, governed mostly by turnover, i.e., the replacement of disappearing species by new emerging species along the gradient [19]. Furthermore, even higher functional beta diversity was expected, because the trait compositions of diatom communities are typically strongly affected by environmental gradients [16]. 3) *Is the functional beta diversity affected by species loss in the ecosystem?* The functional beta diversity of communities was expected to increase following species loss, indicating weaker resilience and higher variability in the communities [2].

## Methods

### Study Area and Sampling

The study area is located on the Finnish coast of the Baltic Sea, close to the Hanko-peninsula, and stretches from the river Karjaanjoki, through its estuary Pojo Bay, to the archipelago area and finally the open sea (see Fig. 2). The river Karjaanjoki is a lowland river characterized by slow river flow. Its estuary, Pojo Bay, is a fjord-like estuary, separated from the archipelago zone by a 6-m deep sill. The archipelago zone is generally shallow, below 40 m, and characterized by a mosaic of islands and islets. It transforms gradually to the open Gulf of Finland. Together, this area represents a salinity gradient from 0 to 5–7, which leads to communities changing from species with a freshwater origin to species with a more marine origin. The effect of river water extends approximately 20 km from the river mouth, and the outer archipelago is a mixing region of several water masses from Pojo Bay as well as from the open Gulf of Finland. Thus, biotic communities are a mixture of brackish and fresh water species. The entire gradient encompasses ca. 60 km.

The sampling was conducted over a short time period in mid-summer, 26 June–4 July 2017, to minimize the potential for temporal variability. A total of 51 samples were collected from as many sites with high variability of environmental variables, such as salinity, exposure to waves and nutrients. Sampling was conducted following the recommendations by Kelly et al. [21]. At each site, ten cobble-sized stones were randomly selected along the shoreline from depths of 20–50 cm. The biofilm was collected by scraping the surfaces of stones with a toothbrush (25 cm^2^ per stone) and the accumulated suspension was pooled into a composite sample. After sampling, the diatom samples were stored in cold (+ 4 °C) and dark conditions until further analyses. To account for the stability of the substrate, which may affect the growing conditions of diatoms, the dimensions (length ⨯ width ⨯ height) of each sampling stone were measured. Salinity, pH, and water temperature were also measured in situ, and water samples were collected from each site. The water samples were frozen immediately after sampling and later used for nutrient analyses.

### Laboratory Analyses and Wind Exposure Calculations

The diatom samples were boiled with hydrogen peroxide (30% H_2_O_2_) to remove organic material, and the cleaned diatoms were mounted on slides using Naphrax (Brunel Microscopes Ltd., UK). Then, a phase contrast light microscope with a × 1000 magnification was used to identify 500 valves per sample to the lowest possible taxonomic level (typically species level) following Krammer and Lange-Bertalot [22–25], Snoeijs [26], Snoeijs and Vilbaste [27], Snoeijs and Potapova [28], and Snoeijs and Kasperovicienè [29]. To account for the functional composition of communities, the abundances of traits were used, because they are robust indicators of ecological strategy. Diatom species were classified according to their size (biovolume classes: large > 1000 μm^3^/small < 1000 μm^3^), mobility (mobile/non-mobile), type of attachment (adnate/pedunculate [which was further divided to pad-attached/stalk-attached]/non-attached), colonization (colonial/non-colonial), growth form (low-profile/high-profile/motile/planktonic) [30], and nitrogen-fixing abilities (nitrogen-fixer/non-nitrogen-fixer) [31]. To identify traits for each diatom species, in addition to above mentioned species and trait literature, Diatoms of North America [32] was used.

The nutrient analyses of the water were conducted with an automated photometric analyzer (Thermo Scientific Aquakem 250 [Thermo Fisher Scientific Oy, Vantaa, Finland]) (total P, NO_2_^−^ + NO_3_^−^, PO_4_^3−^, and Si), except for total N and NH_4_^+^, which we analyzed manually. Flow velocity at sites was not measured, but to account for the wind exposure of our sampling sites, fetch, i.e., the distance over which wind can travel across open water, was calculated. This was done by means of a transparent circular disc divided into 40 lines, 9° apart from each other. The center of the disc was placed on a nautical sea chart at the exact study site, and distance to next shore, island, or islet along every line was calculated. Thus, 40 values for each site were obtained. As a measure of fetch of the site, the average of these 40 values was used [modified from 33].

### Statistical Analyses

Prior to statistical analyses, NO_2_^−^ + NO_3_^−^, NH_4_^+^, PO_4_^3−^, Si, total P, total N, salinity, and fetch values were log10-transformed to reduce their skewed distributions. Statistical dependence between the explanatory environmental variables was assessed using Pearson’s correlation coefficients. All pairwise correlations were *r* ≤ |0.7|; therefore, all variables were retained for further analyses. Environmental variables were visualized using R package ggplot2 [34], where regressions were computed using function *geom_smooth()* with method = “loess,” which computes a smooth local regression. To enable comparison in the visualization, all variables were standardized between 0 and 1.

#### The Effect of Environment on Taxonomic and Functional Beta Diversity

To study the effects of individual environmental variables on taxonomic and functional dissimilarity, generalized dissimilarity modeling (GDM) was used [35]. GDM is a method designed for studying the importance of predictor variables for beta diversity by modeling spatial variation of community composition between pairs of geographical locations. GDMs with species and trait abundance data were performed using Bray-Curtis distance for species data and Gower distance for trait data. Function *formatsitepair* was used to convert the response and predictor variable data to a site-pair table, and function *gdm.varImp* with 50 permutations to estimate *p* values for the whole model and each of the predictor variables. GDMs were constructed using R package gdm [36].

#### Taxonomic and Functional Beta Diversity

Similarities in community composition between the first sampling site and each of the other sites were calculated. First, similarities in taxonomic composition with abundance data were calculated using the Bray-Curtis similarity index [37]. Communities are often characterized by a high number of rare species, which can lead to noise in the analyses [38]. To avoid this, Bray-Curtis similarity indices were also calculated with only widely distributed species, i.e., species that were present at more than 50% of sites. Due to different numbers of species (up to 87) and traits (up to 8) per site, the variation in taxonomic similarity was also quantified using randomly sampled subsets of 8 species. Random sampling was conducted in R using function *sample()*. To be able to indicate the possible effect of species richness gradients on the dissimilarity, similarity in species composition was then assessed with Simpson similarity index [39], which uses presence-absence data and is independent of species richness. Finally, the Bray-Curtis similarity index was used to study the similarity in functional abundance, i.e., diatom traits. Considering both taxonomic and functional diversity can show contrasting patterns and give different perspectives on processes structuring communities [19] and on the effect of community diversity on ecosystem functioning [18]. Bray-Curtis and Simpson similarity indices range from 0 to 1, 1 representing total similarity and 0 representing total dissimilarity. Similarity indices were calculated with R package vegan [40].

To visualize taxonomic and functional beta diversity along our sampling gradient, Bray-Curtis similarity values were interpolated. This was done with QGIS using Inverse Distance Weighting. Map layers were derived from the databases of European Environment Agency, Paituli Spatial Data Service, and SYKE.

To quantify the variation in similarity, a distance-based approach was used [41]. Instead of using all pairwise comparisons, the community similarities were plotted against geographical distance (km) from site 1 and the significance of this distance-decay relationship was analyzed by using linear models. Adjusted *R*^2^ was used as coefficient of determination, and *p* values were calculated using the *F* test [42]. Linear models were plotted using R package ggplot2 [34]. Halving distances, i.e., distances that halve the similarity from its value at 1-km distance, were also calculated as described by Soininen et al. [43] for taxonomic and functional composition using abundance data.

Then, all pairwise relationships between taxonomic and functional similarities and environmental heterogeneity were examined. Similarities were counted with a framework, where the total beta diversity is the sum of beta diversity explained by replacement of species/traits and beta diversity explained by species/trait richness differences. Functional beta diversity was calculated based on a functional tree that depicts species relationships [44]. For the similarities, Sørensen similarity index with abundance data was used. Environmental heterogeneity was counted as Euclidean distance of scaled variables. These were done with R packages BAT [44], cluster [45], and vegan [40].

To analyze different perspectives of diversity, the taxonomic and functional beta diversities of the whole sampling area were divided to turnover and nestedness components. The portion of turnover was counted using formula

$$ \frac{\mathrm{value}\ \mathrm{of}\ \mathrm{turnover}\ \mathrm{component},\mathrm{measured}\ \mathrm{as}\ \mathrm{Simpson}\ \mathrm{dissimilarity}}{\mathrm{value}\ \mathrm{of}\ \mathrm{overall}\ \mathrm{beta}\ \mathrm{diversity},\mathrm{measured}\ \mathrm{as}\ \mathrm{Sorensen}\ \mathrm{dissimilarity}} $$

and the portion of nestedness using formula

$$ \frac{\mathrm{value}\ \mathrm{of}\ \mathrm{nestedness}\ \mathrm{component},\mathrm{measured}\ \mathrm{as}\ \mathrm{nestedness}-\mathrm{resultant}\ \mathrm{fraction}\ \mathrm{of}\ \mathrm{Sorensen}\ \mathrm{dissimilarity}}{\mathrm{value}\ \mathrm{of}\ \mathrm{overall}\ \mathrm{beta}\ \mathrm{diversity},\mathrm{measured}\ \mathrm{as}\ \mathrm{Sorensen}\ \mathrm{dissimilarity}} $$

This was done with the R package betapart [46].

#### The Effect of Species Loss on Functional Beta Diversity

To account for the effect of species loss on beta diversity, all pairwise relationships between taxonomic similarities and environmental heterogeneity, and functional similarities and environmental heterogeneity, were examined using the functional tree approach (see above for details) with randomly sampled subsets of species instead of full communities. To mimic species loss, 5–95% of species were randomly sampled, and the model was run 100 times with each randomly sampled subset. To analyze the functional similarities of each subset, the mean of the slopes was calculated, and the significance of differences between subsample sizes was estimated using analysis of variance (ANOVA). This statistical method for simulating species loss fails to consider some aspects that are present in real-world communities, such as biotic interactions and differences in species’ sensitivity to environmental change [47]. However, these aspects are largely unknown in microbial communities, which prevents their inclusion in models. Thus, this approach gives the currently best possible idea on the effects of species loss in diatom communities.

All statistical analyses were calculated using R version 3.5.3 [48].

## Results

**Fig. 1:**
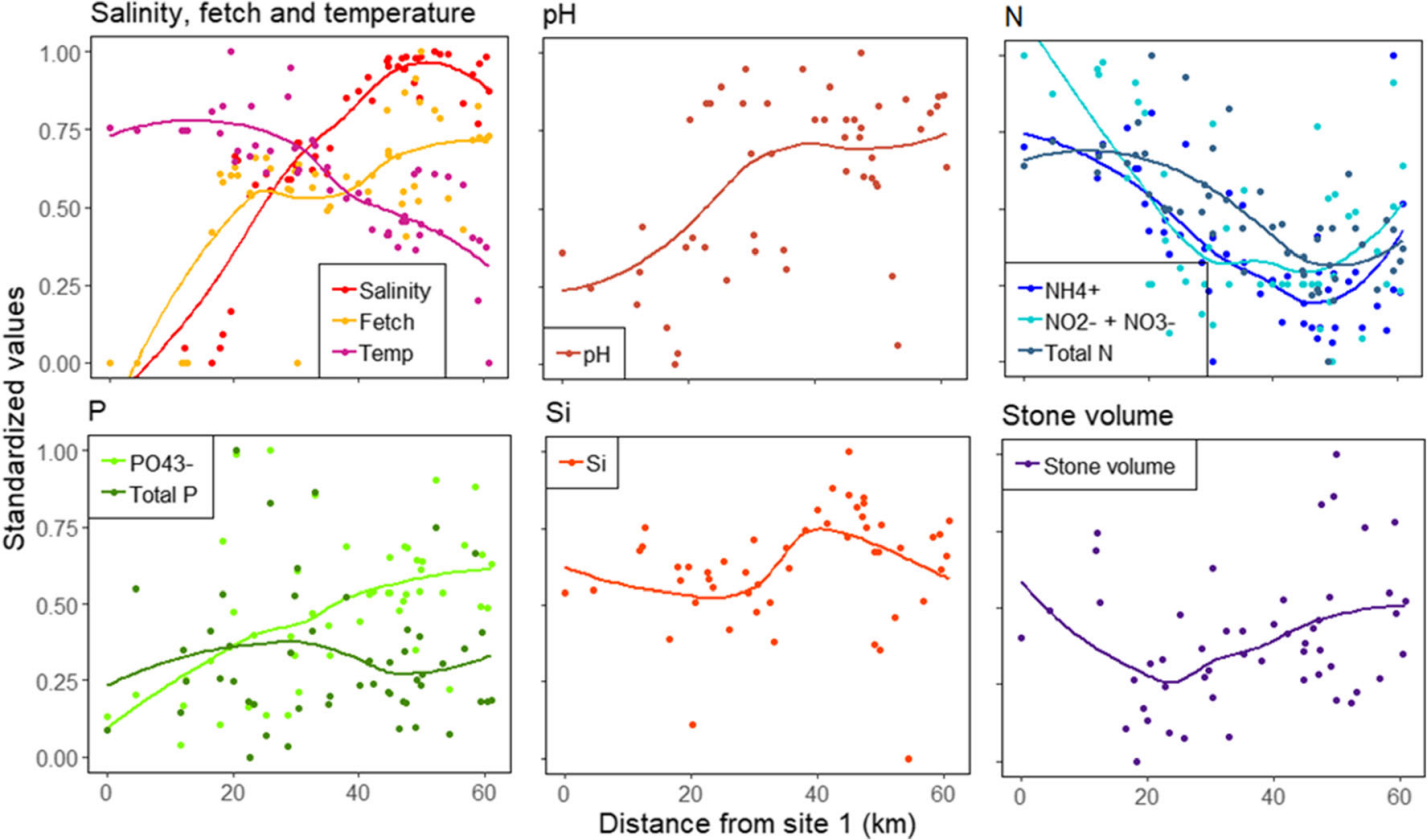
Variation in environmental variables along the sampling gradient. Site 1 is located in the river and site 51 in the open sea. Lines denote smooth local regressions. N denotes nitrogen, P phosphorus, and Si silicate

Total species richness in the samples was 408, of which 33 species were present in more than 50% of the sites and were thus considered widely distributed. Species richness per site varied between 26 and 87 (Appendix Table 1). All the traits that were accounted for were present at every site, except planktonic life forms that were absent from six sites in the archipelago close to the open sea (Appendix Table 2). Environmental conditions varied considerably along the geographical gradient (Fig. 1). From site 1 (freshwater) to site 51 (marine), salinity, fetch, pH, and PO_4_^3−^ showed an increasing trend, temperature, NO_2_^−^ + NO_3_^−^, NH_4_^+^, and total N showed a decreasing trend, stone volume showed a unimodal trend, and Si and total P showed variable trends.

### The Effect of Environment on Taxonomic and Functional Beta Diversity

Taxonomic dissimilarity was best explained by fetch, salinity (*p* < 0.001) and temperature (*p* = 0.040) (Table 1). The model explained 61.40% of deviance and was highly significant (*p* < 0.001). Functional dissimilarity was best explained by temperature (*p* < 0.001) (Table 1). The model explained 15.78% of deviance and was highly significant (*p* < 0.001).

**Table 1:** Results of the generalized dissimilarity modeling (GDM) to explain taxonomic and functional dissimilarity. 0.001 ***, < 0.01 **, < 0.05 *, < 0.1

Deviance explained	Diatom species		Diatom traits	
	61.40%		15.78%	
Model *p* value	< 0.001***		< 0.001***	
	Importance	*p* value	Importance	*p* value
Fetch	17.598	0.00***	0.398	0.60
Salinity	9.204	0.00***	6.039	0.10
Temperature	2.678	0.04*	37.202	0.00***
Total N	1.342	0.18	2.620	0.22
Total P	0.689	0.24	0.856	0.48
Stone volume	0.421	0.26	1.826	0.32
NO_2_^−^ + NO_3_^−^	0.413	0.28	0.897	0.44
pH	0.164	0.40	15.211	0.12
PO_4_^3−^	0.129	0.44	0.000	0.96
Si	0.045	0.60	0.323	0.64
NH_4_^+^	0.069	0.72	0.000	1.00

### Taxonomic and Functional Beta Diversity

**Fig. 2:**
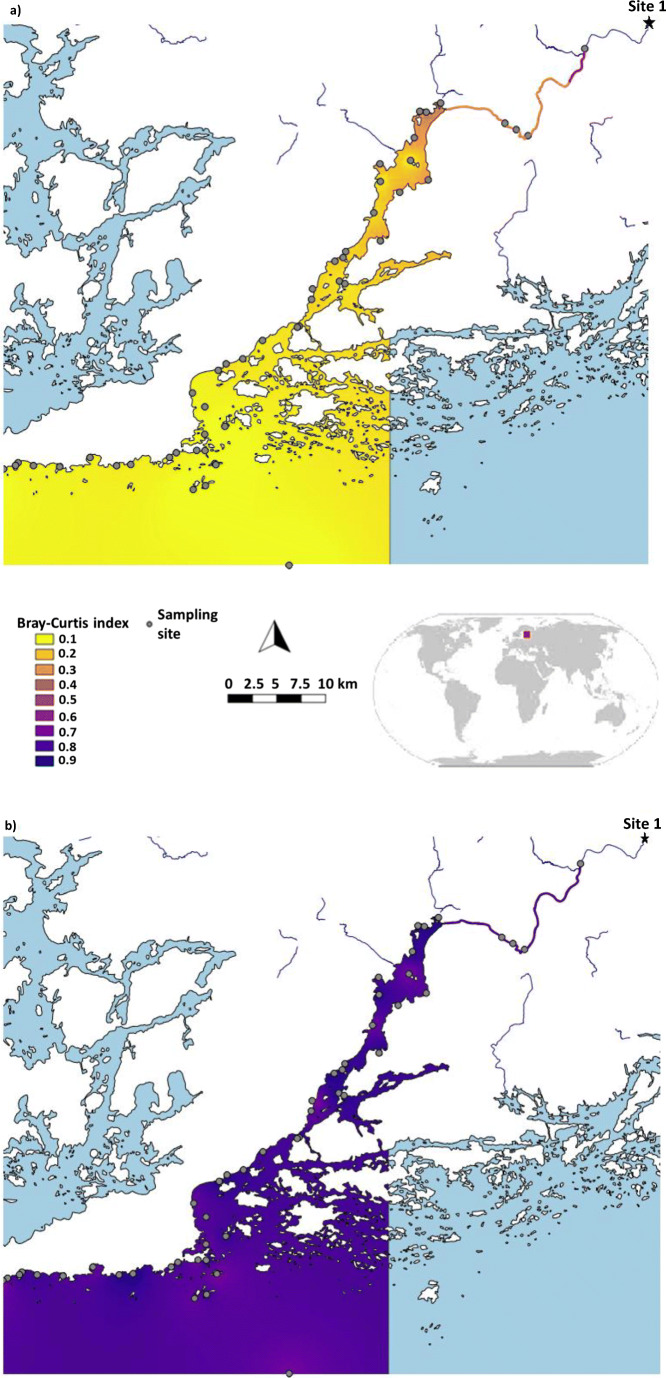
Interpolated similarity between site 1 and the study area (Bray-Curtis index values, abundance data) and (a) species demonstrating a decreasing gradient of community similarities from the river to the open sea and an overall low similarity of communities, and (b) traits demonstrating high similarity of communities

**Fig. 3:**
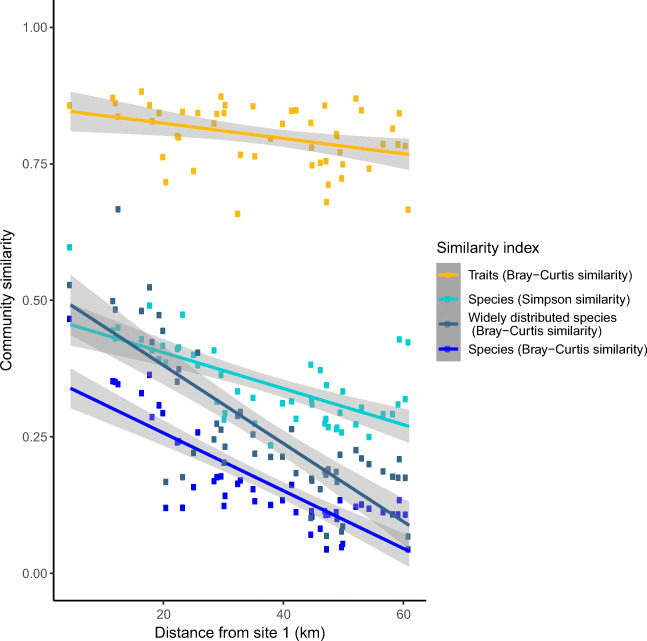
The relationships between community similarity and geographical distance (km). Points represent similarity between the first sampling site and each of the other sites. Different aspects of taxonomic similarities are visualized with blue points and lines, and functional similarity is visualized with yellow points and line. Functional community similarity remains high during the whole geographical distance, whereas taxonomic community similarity becomes small towards the end of the geographical gradient

The compositional similarities between the first sampling site and each of the other sampling sites showed differences between species and trait data (Figs. 2 and 3). Similarity between the first and last sites was low concerning any aspect of species composition: (i) Bray-Curtis similarity index with abundances of all species was low (0.044), and the significant linear model suggested decreasing trend between similarity and geographical distance (*R*^2^ 0.690, *p* < 0.001); (ii) Bray-Curtis similarity index with abundances of dominant species was also low (0.067), and the significant linear model showed a decreasing trend (R^2^ 0.639, *p* < 0.001); similar results were observed with randomly sampled subsets of 8 species (detailed results not shown here); (iii) Simpson similarity index with presence-absence data of all species was also low (0.234), and the linear model was decreasing and significant (*R*^2^ 0.434, *p* < 0.001). In contrast, similarity for trait abundance was high: Bray-Curtis similarity index with abundances of traits was high (0.658), and the slope of the linear model was only slightly decreasing (*R*^2^ 0.121, *p* 0. 008).

**Fig. 4:**
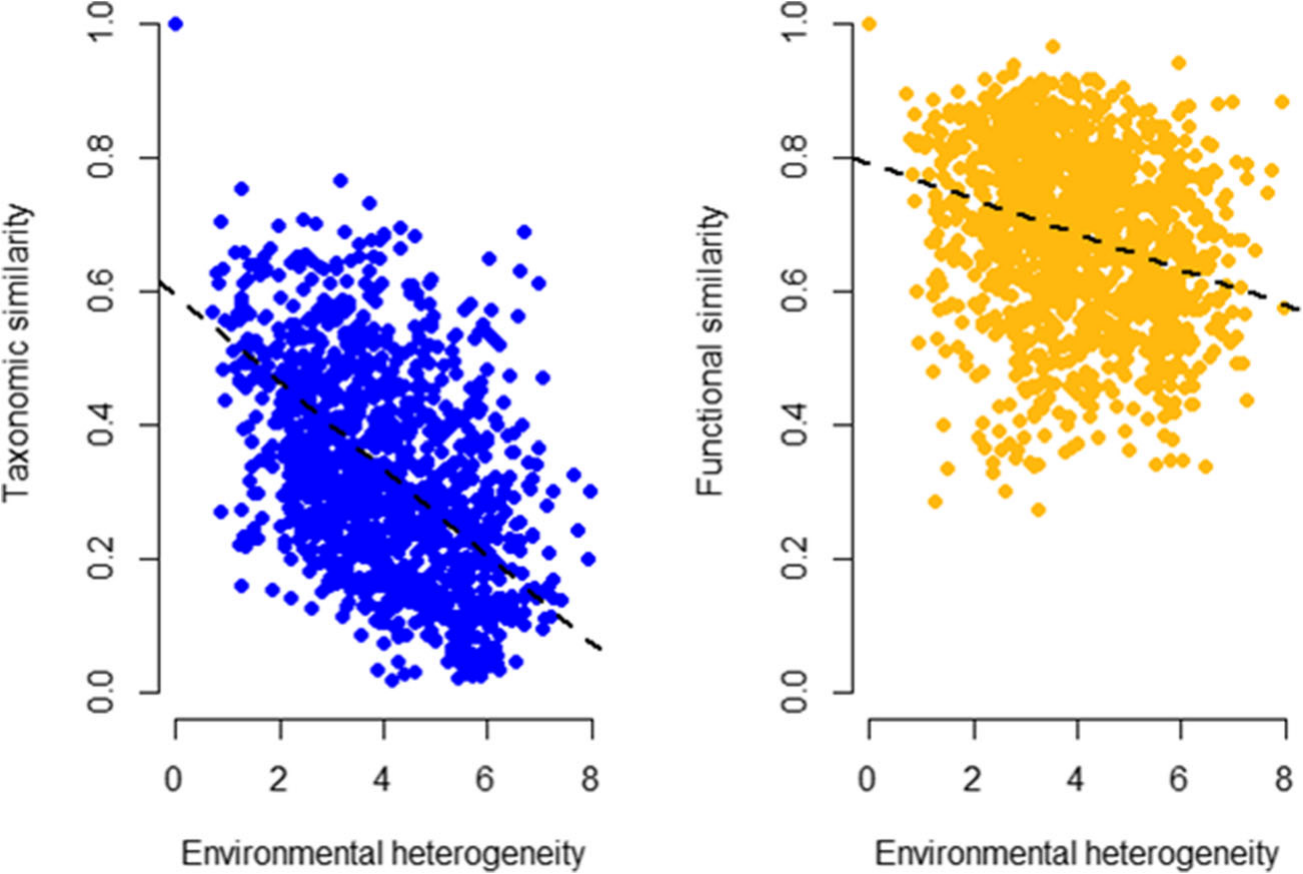
Pairwise relationships between taxonomic and functional similarities (Sørensen similarity index, abundance data) and environmental heterogeneity (Euclidean distance of standardized variables, i.e., the cooperation of all the environmental predictor variables). The dashed line denotes linear model fitted to data

Relationships between pairwise similarities and environmental heterogeneity also showed differences between taxonomic and functional data (Fig. 4). For the taxonomic data, the mean similarity was 0.339 (minimum 0.017, maximum 1.000). For the functional data, the mean similarity was 0.688 (minimum 0.274, maximum 1.000). Halving distance was 39 km for the taxonomic data and 98 km for the functional data suggesting higher turnover of taxonomic composition.

Taxonomic beta diversity was almost completely (98.0%) due to turnover, i.e., replacement of species by others along the gradient. Nestedness accounted for only 2.0% of taxonomic beta diversity. For the functional beta diversity, turnover was only slightly more important (54.2%) than nestedness.

### The Effect of Species Loss on Functional Beta Diversity

Pairwise relationships between functional similarity and environmental heterogeneity, using randomly sampled subsets of species, showed that the slopes of randomly sampled 5–45% of species were significantly more negative than the slope of 100% of species. Pairwise relationships between taxonomic similarity and environmental heterogeneity showed that the slopes of randomly sampled 5–15% of species were significantly more negative than the slope of 100% of species (Table 2).

**Table 2:** Slopes of pairwise relationships between taxonomic and functional similarities (Sørensen similarity index, abundance data) and environmental heterogeneity (Euclidean distance of standardized variables). Percentage of species denotes randomly sampled subsets. < 0.001***, < 0.01 **, < 0.05 *, < 0.1

% of species	Taxonomic	Functional	% of species	Taxonomic	Functional
	Slope (mean	Slope (mean)		Difference between slopes (*p* value)	Difference between slopes (*p* value)
100	− 0.06513	− 0.02666	100–95	1.000	1.000
95	− 0.06527	− 0.02707	100–90	1.000	1.000
90	− 0.06493	− 0.02715	100–85	1.000	1.000
85	− 0.06439	− 0.02685	100–80	1.000	1.000
80	− 0.06393	− 0.02680	100–75	1.000	1.000
75	− 0.06501	− 0.02856	100–70	1.000	1.000
70	− 0.06361	− 0.02817	100–65	1.000	0.440
65	− 0.06503	− 0.03088	100–60	1.000	0.251
60	− 0.06514	− 0.03132	100–55	1.000	0.639
55	− 0.06396	− 0.03048	100–50	0.985	0.537
50	− 0.06291	− 0.03069	100–45	1.000	0.001**
45	− 0.06417	− 0.03380	100–40	0.975	0.002**
40	− 0.06280	− 0.03366	100–35	1.000	< 0.001***
35	− 0.06383	− 0.03487	100–30	1.000	0.000***
30	− 0.06425	− 0.03676	100–25	1.000	0.000***
25	− 0.06372	− 0.03674	100–20	0.549	0.000***
20	− 0.06171	− 0.03842	100–15	0.008**	0.000***
15	− 0.05967	− 0.03933	100–10	< 0.001***	0.000***
10	− 0.05771	− 0.03953	100–5	< 0.001***	0.000***
5	− 0.05761	− 0.04499			

## Discussion

To our knowledge, this study is one of the first to address the functional beta diversity of microorganisms in estuarine or marine environments. It has previously been shown that the functional alpha diversity of marine microorganisms, namely benthic diatoms, is tightly linked to ecosystem functioning [18]. Because beta diversity is closely related to the connectivity of communities and consequently to ecosystems’ resilience and recovery potential after disturbance, expanding research to beta diversity is important in present times of global change.

### The Effect of Environment on Taxonomic and Functional Beta Diversity

A common approach in studies on macroorganisms is the partitioning of mechanisms behind beta diversity patterns [49]. Possible mechanisms include deterministic processes, such as environmental filtering, or historical and present-day large-scale processes, such as speciation or limited dispersal. However, due to the lack of consensus on general assembly rules of microorganisms [50], it is unclear whether these theories apply to microorganisms. Thus, to avoid uncertainties, the study area used here exhibits an exceptionally steep environmental gradient along a short geographical distance (~ 60 km from the first sampling site to the last). Due to the short distance and continuous water flow between sites, deterministic processes, namely environmental filtering, are probably most likely to control beta diversity in the study area. In fact, measured environmental variables explained 61.4% of our taxonomic beta diversity. This is a high value in studies concerning microorganisms, because rare species usually lead to high amounts of noise [38]. It is speculated that the unexplained part of variation may be due to unmeasured environmental variables, such as habitat characteristics, or bias caused by unintentional differences in sampling or sample processing.

In contrast to predictions, taxonomic beta diversity in the study was mostly controlled by, not only salinity which usually is assumed to be the controlling factor in estuarine environments [e.g., 20], but also by the other variables that represent the transition from freshwater environment to marine realm, namely wind exposure and temperature. The environmental conditions were also highly variable in terms of nutrients, pH, and stability of substrate, and as a result, taxonomic beta diversity was reasonably high already between the first adjacent sites. This variation in environment also led to very high total species richness (408 species) in the study area. In contrast, the functional composition was much more robust to variations in the environment, and only 15.8% of deviance was explained by the environment. This was an interesting finding, because in freshwater habitats the functional composition of diatom communities has been found to be more sensitive to environmental gradients than the taxonomic composition [16]. Temperature was the only variable that significantly explained variation in the functional composition of communities. Although temperature may have an effect on communities per se, it may also have been correlated with some unmeasured variables, such as biotic interactions or light intensity.

### Taxonomic and Functional Beta Diversity

As expected, a strong change in the environment resulted in almost complete taxonomic turnover of benthic diatoms. Such high beta diversity is common in the presence of dispersal limitation [e.g. 51], but rare in areas like the one presented here, where species compositions are the result of environmental filtering [e.g., 19]. Taxonomic beta diversity was high even when the effects of variation in species richness and the noise due to rare species were accounted for by using only dominant species or randomly sampled subsets of species. The taxonomic beta diversity was mainly (98.0%) governed by turnover, i.e., replacement of disappearing species by new emerging species along the gradient, which is a typical finding in field studies [52].

In contrast, functional beta diversity was low and almost evenly distributed among turnover and nestedness. Thus, the diatom communities stayed functionally almost similar despite large changes in species composition and environmental variables, and species were replaced by taxonomically new but functionally almost similar species. The high functional similarity may partly stem from similar biotic interactions, e.g., grazing pressure, throughout the entire estuary. Similar results, demonstrating high taxonomic but low functional beta diversity, have been found in some studies on macroorganisms [19], but it has usually been linked to dispersal limitation or historical legacy effects [6]. Although it is recognized that the high taxonomic but low functional beta diversity may partly stem from the high number of taxonomic species versus considerably lower number of functional traits, it is noteworthy that the result of high taxonomic but low functional beta diversity remained similar even when the strong difference between the amount of species and traits was accounted for by using only widely distributed species. The results show that microorganisms are most likely able to maintain functional similarity even in the presence of high environmental variability. Although the number of traits available for microbial organisms is limited and fails to cover all the functions of an ecosystem, the results may also indicate that, through high biodiversity and consequent redundancy in functions, microorganisms exhibit a strong insurance effect against environmental change. Furthermore, because the functional traits used in this study represent morphological characteristics that are tightly linked to ecosystem functions, such as biomass production and trophic webs, high functional similarity of diatom communities also possibly suggests an insurance effect for stable ecosystem functioning.

### The Effect of Species Loss on Functional Beta Diversity

The results, however, show that decreasing taxonomic diversity has a strong impact on the functional beta diversity of diatom communities, as the functional similarity of the communities showed a significant reduction, when the total species richness was decreased to 45% of the current richness. This suggests that changes in the environmental conditions can significantly decrease the functional similarity and connectivity of communities, leading to differently functioning local communities at different parts of the region. This may increase the functional vulnerability and decrease the resilience and recovery potential of benthic communities [8]. Thus, changing environment and consequently decreasing taxonomic diversity may affect ecosystem functioning and services provided by these communities. It should be noted that the method used here for analyzing the effect of decreasing species richness on functional similarity was statistically removing species from communities. This method fails to consider aspects present in real-world communities, such as biotic interactions and differences in species’ sensitivity to environmental change. Thus, controlled field or laboratory experiments would be valuable for further confirming the generality of the results presented here and to increase their relevance for real-world ecosystems.

However, the findings of changing functional diversity during changes in the environment or in the taxonomic composition of communities are consistent with Teixidó et al. [2], who concluded that even moderate disturbance effects (ocean acidification) can have significant impacts on the functional diversity and redundancy of benthic macroorganisms, and with D’agata et al. [53], who found that the functional characteristics of coral reef fishes are highly vulnerable to species loss due to fishing. These findings indicate that while some functional traits are abundant and redundant in communities, other traits are rare and sensitive to species loss [53]. In benthic diatoms, disappearing of these sensitive traits may lead to communities with decreased resource acquisition and tolerance to stressors and consumers [54], which may result in lower productivity. This emphasizes the importance of biodiversity for the stable functioning of benthic communities.

## Electronic Supplementary Material

ESM 1 (XLSX 31 kb)

ESM 2 (XLSX 38 kb)

ESM 3 (TXT 68 kb)

## Data Availability

Raw data that support the results of this article is available as Appendix Tables 1 and 2.
